# Supplementary material for: Impact of a New Preoperative Stratification Based on Cardiac Structural Compromise in Patients with Severe Aortic Stenosis Undergoing Valve Replacement Surgery
Source: Diagnostics (Basel). 2024 Oct 9;14(19):2250. doi: 10.3390/diagnostics14192250 (PMC11475271; doi:10.3390/diagnostics14192250)
Supplement: Supplementary file 1 [file diagnostics-14-02250-s001.zip › diagnostics-3231470-supplementary.pdf]

Supplementary Materials

Table S1. Univariate analysis of composite outcome predictors of death and hospital readmission – Page 2

Table S2. Multivariate analysis models of predictors for primary outcomes – Page 3

Table S3. Multivariate analysis models of predictors for primary outcome with original classification – Page 4

Table S4. ROC analysis of short-term follow-up time. – Page 6

Table S5. ROC analysis of complete follow-up time.

Table S1. Univariable analysis of composite outcome predictors of death and hospital readmission

	HR (95% CI)	p
**Clinical data**		
**Sex**	1.10 (0.64-1.88)	0.728
**Age (Years)**	1.01 (0.99-1.03)	0.240
**Diabetes mellitus**	1.09 (0.63-1.88)	0.740
**Hypertension**	1.18 (0.67-2.06)	0.564
**Coronary artery disease**	1.37 (0.76-2.48)	0.286
**Number of vessels connected**	1.15 (0.79-1.68)	0.450
**EuroSCORE II. %**	1.10 (1.04-1.16)	**<0.001**
**Symptoms**		
**Functional class (NYHA)**	0.80 (0.52-1.24)	0.332
**Laboratory data**		
**Clearance de creatinina. ml/min**	0.98 (0.96-0.99)	**0.042**
**Hemoglobin. g/dl**	0.84 (0.73-0.98)	**0.028**
**Echocardiographic data**		
**Interventricular septum. mm**	0.97 (0.85-1.10)	0.675
**Back wall. mm**	0.98 (0.84-1.16)	0.885
**Ventricular mass. g/m2**	1.00 (0.99-1.01)	0.214
**Transaortic mean gradient. mmHg**	0.97 (0.95-0.99)	**0.021**
**Area valvar aórtica. cm^2**	2.26 (0.39-13.15)	0.362
**Aortic Insufficiency**	1.31 (0.64-2.69)	0.456
**Left ventricular hypertrophy**	1.27 (0.59-2.69)	0.533
**Diastolic dysfunction**	0.88 (0.49-1.58)	0.678
**Left atrial enlargement**	2.86 (1.43-5.70)	**0.003**
**Moderate/severe secondary mitral regurgitation**	2.27 (1.23-4.19)	**0.008**
**Atrial fibrillation**	4.17 (2.02-8.63)	**<0.001**
**Moderate/severe tricuspid regurgitation**	2.40 (1.23-4.67)	**0.010**
**Moderate/severe right ventricular dysfunction**	2.70 (0.84-8.71)	0.095
**Pulmonary systolic pressure. mmHg**	1.04 (1.00-1.07)	0.015
**Ejection fraction. %**	0.96 (0.94-0.98)	**<0.001**
**Adapted Généreux classification**	2.47 (1.69- 3.60)	**<0.001**

NYHA: New York Heart Association. AF: Atrial fibrillation

Table S2. Multivariable analysis models of predictors for primary outcome

UNIVARIABLE					MULTIVARIABLE				
	HR	95.0% CI		p		HR	95.0% CI		p
**AGE**	1.021	1.004	1.039	.015	AGE	0.998	0.971	1.025	0.869
**BNP**	1.000	1.000	1.001	<0.001	BNP	1.000	1.000	1.000	0.286
**Mean gradient**	0.975	0.963	0.987	<0.001	Mean gradient	0.985	0.962	1.010	0.243
**NYHA**	2.139	1.666	2.745	<0.001	NYHA	1.138	0.746	1.738	0.548
**Clearance creatinine**	0.980	0.971	0.989	<0.001	Clearance creatinine	1.004	0.989	1.020	0.599
**Proposed classification**	3.217	2.552	4.056	<0.001	Proposed classification	2.612	1.705	4.003	<0.001
**EuroSCORE II**	1.245	1.165	1.331	<0.001	EuroSCORE II	0.949	0.779	1.157	0.607
**Hemoglobin**	0.861	0.786	0.942	.001	Hemoglobin	0.877	0.764	1.006	0.061
**Aortic valve area**	3.580	1.099	11.660	.034		4.267	0.532	34.233	0.172
		**Lower**	**Superior**				**Lower**	**Superior**	
**AGE**	1.021	1.004	1.039	0.015	AGE	0.999	0.971	1.028	0.946
**BNP**	1.000	1.000	1.001	<0.001	BNP	1.000	1.000	1.000	0.423
**Mean gradient**	0.975	0.963	0.987	<0.001	Mean gradient	0.989	0.965	1.013	0.352
**NYHA**	2.139	1.666	2.745	<0.001	NYHA	1.075	0.712	1.623	0.732
**Clearance**	0.980	0.971	0.989	<0.001	Creatinine Clearance	1.005	0.990	1.021	0.505
**Proposed classification**	3.217	2.552	4.056	<0.001	Proposed classification	2.618	1.703	4.025	<0.001
**Hemoglobin**	0.861	0.786	0.942	0.001	Hemoglobin	0.867	0.755	0.996	0.044
**STS SCORE score**	1.328	1.195	1.475	<0.001	STS SCORE	0.975	0.799	1.190	0.807
**Aortic valve area**	3.580	1.099	11.660	0.034		5.730	0.766	42.864	0.089
		**Lower**	**Superior**				**Lower**	**Superior**	
**AGE**	1.021	1.004	1.039	0.015	AGE	0.995	0.969	1.021	0.707
**BNP**	1.000	1.000	1.001	<0.001	BNP	1.000	1.000	1.000	0.271
**EuroSCORE II**	1.245	1.165	1.331	<0.001	EuroSCORE II	0.914	0.757	1.104	0.351
**Mean gradient**	0.975	0.963	0.987	<0.001	Mean gradient	0.976	0.956	0.996	0.022
**Creatinine Clearance**	0.980	0.971	0.989	<0.001	Creatinine Clearance	1.004	0.988	1.020	0.610
**Hemoglobin**	0.861	0.786	0.942	0.001	Hemoglobin	0.873	0.760	1.002	0.053
**NYHA**	2.139	1.666	2.745	<0.001	NYHA	1.189	0.778	1.817	0.424
**Proposed classification**	3.217	2.552	4.056	<0.001	Proposed classification	2.597	1.706	3.952	<0.001
		**Lower**	**Superior**				**Lower**	**Superior**	
**AGE**	1.021	1.004	1.039	0.015	AGE	0.996	0.968	1.024	0.776
**BNP**	1.000	1.000	1.001	<0.001	BNP	1.000	1.000	1.000	0.436
**STS SCORE**	1.328	1.195	1.475	<0.001	STS SCORE	0.966	0.786	1.187	0.741
**Mean gradient**	.975	.963	.987	<0.001	Mean gradient	0.977	0.957	0.998	0.032
**Creatinine Clearance**	.980	.971	.989	<0.001	Creatinine Clearance	1.006	0.991	1.022	0.406
**Hemoglobin**	.861	.786	.942	0.001	Hemoglobin	0.865	0.752	0.994	0.041
**NYHA**	2.139	1.666	2.745	<0.001	NYHA	1.095	0.724	1.656	0.668
**Proposed classification**	3.217	2.552	4.056	<0.001	Proposed classification	2.554	1.673	3.899	<0.001

Table S3. Multivariable analysis models of predictors for primary outcome with original and proposed classification

	HR	95.0% CI		p
**AGE**	1.047	0.986	1.111	0.138
**BNP**	1.000	1.000	1.001	0.069
**EuroSCORE II**	0.839	0.583	1.208	0.347
**STS SCORE**	0.955	0.698	1.306	0.774
**Mean gradient**	0.953	0.919	0.988	0.010
**Creatinine Clearance**	0.987	0.958	1.016	0.368
**NYHA**	0.987	0.424	2.299	0.975
**Hemoglobin**	0.842	0.650	1.091	0.193
**Original classification**	2.123	1.158	3.894	0.015
		**Lower**	**Superior**	
**AGE**	1.041	0.982	1.104	0.175
**BNP**	1.000	1.000	1.001	0.102
**EuroSCORE II**	0.836	0.576	1.213	0.346
**STS SCORE**	0.953	0.701	1.294	0.757
**Mean gradient**	0.958	0.923	0.994	0.022
**Creatinine Clearance**	0.987	0.958	1.016	0.366
**NYHA**	0.876	0.363	2.116	0.769
**Hemoglobin**	0.864	0.669	1.116	0.263
**Proposed classification**	3.435	1.552	7.604	0.002
		**Lower**	**Superior**	
**AGE**	1.051	0.987	1.119	0.120
**BNP**	1.000	1.000	1.001	0.119
**EuroSCORE II**	0.860	0.610	1.214	0.392
**STS SCORE**	0.978	0.715	1.339	0.891
**Mean gradient**	0.955	0.923	0.989	0.010
**Creatinine Clearance**	0.982	0.954	1.010	0.210
**NYHA**	1.036	0.455	2.360	0.933
**Original classification**	2.019	1.115	3.656	0.020
		**Lower**	**Superior**	
**AGE**	1.047	0.986	1.112	0.130
**BNP**	1.000	1.000	1.001	0.179
**EuroSCORE II**	0.865	0.612	1.222	0.411
**STS SCORE**	0.961	0.706	1.309	0.803
**Mean gradient**	0.962	0.928	0.996	0.029
**Creatinine Clearance**	0.983	0.955	1.012	0.242
**NYHA**	0.918	0.390	2.158	0.844
**Proposed classification**	3.455	1.536	7.774	0.003

		Lower	Superior	
AGE	1.036	0.982	1.092	0.193
BNP	1.000	1.000	1.001	0.077
Mean gradient	0.953	0.921	0.987	0.007
Creatinine Clearance	0.992	0.967	1.018	0.542
Hemoglobin	0.907	0.701	1.174	0.459
NYHA	0.972	0.463	2.037	0.939
Original classification	1.859	1.046	3.305	0.035
		Lower	Superior	
AGE	1.033	0.981	1.089	0.218
BNP	1.000	1.000	1.001	0.140
Mean gradient	0.959	0.926	0.993	0.019
Creatinine Clearance	0.993	0.968	1.018	0.590
Hemoglobin	0.932	0.724	1.198	0.581
NYHA	0.844	0.391	1.819	0.664
Proposed classification	3.185	1.431	7.088	0.005
		Lower	Superior	
AGE	1.040	0.986	1.097	0.148
BNP	1.000	1.000	1.001	0.100
Mean gradient	0.954	0.922	0.987	0.007
Creatinine Clearance	0.988	0.965	1.012	0.341
NYHA	0.997	0.485	2.049	0.993
Original classification	1.830	1.033	3.239	0.038

Table S4. ROC analysis of short-term follow-up time.

Variables				
	**AUC**	**Standard error**	**CI 95%**	**p**
Original classification	0.815	0.039	0.738 – 0.892	<0.001
Proposed classification	0.12	0.041	0.732 – 0.892	<0.001
EuroSCORE II	0.722	0.055	0.614 – 0.830	<0.001
STS score	0.695	0.057	0.583 – 0.807	0.001

Table S5. ROC analysis of complete follow-up time.

Variables				
	**AUC**	**Standard error**	**CI 95%**	**p**
Original classification	0.760	0.035	0.692 – 0.829	<0.001
Proposed classification	0.758	0.036	0.687 – 0.829	<0.001
EuroSCORE II	0.729	0.033	0.663 – 0.794	<0.001
STS score	0.711	0.032	0.649 – 0.773	<0.001
